# Ectopic Pregnancy

**Published:** 1892-12

**Authors:** 


### ECTOPIC PREGNANCY.

Martin, of Berlin, at the recent International Congress at Brussels, gave the results of his observation in 56 cases of ectopic gestation. So far as etiology is concerned, our knowledge scarcely extends beyond a mechanical hindrance to the passage of the ovum from the ovary to the uterus. Ovarian pregnancy has been observed, although very rarely. The most frequent location of the ovum is in the ampulla of the tube. In his case, such was the situation in 49 ; in 34 upon the right side, in 22 upon the left. So soon as the ovum lodges in the tube, the decidua begins an irregular development, never entirely enclosing the ovum. The serotina is less developed than in uterine pregnancy; the fœtal portion of the placenta does not differ from that of normal pregnancy. The muscular layer of the tube does not increase progressively, but after a certain point undergoes excessive atrophy. More or less irritation of the peritoneum is present. The uninvolved portion of the tube undergoes no change except that the fimbrian extremity is closed. As the ovum increases in size, the tube becomes bent and adherent. In 11 of his cases the ovum was attached on the side of the tube next the broad ligament. The uterus increases in volume during ectopic gestation and is in a condition of general hypertrophy. The uterine decidua also develops, but to a less extent than in normal pregnancy. It often undergoes a retrograde process while the ectopic gestation continues its course. Most cases of ectopic pregnancy terminated in tubal abortion within the first three months. Martin's table shows that 15 terminated in the first month, 13 in the second, 11 in the third, 7 in the fourth, the remainder occurring before the ninth month. The fœtus attained viability but once in his cases. The interruption of ectopic pregnancy usually occurs from failure in the physiological conditions existing between the ovum and the cavity containing it. The pain which occurs at tubal abortion is caused by the passage of blood through the tube. The newly developed blood vessels burst, while the strongly developed muscular layer of the tube is not ruptured. Thus many cases result in spontaneous recovery. Convalescence, however, is prolonged, and there is danger of death from shock, hemorrhage, and the development of septic infection. Localized pelvic peritonitis is also common. Martin quotes Schauta's collection of 241 cases in which the ovum was not removed by operation. Of these, 128 ruptured into the abdominal cavity with bleeding; in 22, a hæmatocele formed with peritonitis. In 34, rupture occurred into the intestine; in 9, into the bladder; in 5, the abdominal wall was perforated. In 4, the vagina was entered; in 6, the ovum escaped through the uterus; in 4 cases it became incarcerated with ileum; add in 9 cases lithopædion was formed without especial annoyance to the patient. He had personally observed 5 cases in which rupture occurred, which were not treated by operation; all of them perished. The symptoms of ectopic gestation embrace some of those of normal pregnancy; early in the case, symptoms of peritoneal irritation predominate.

Menstruation is disordered, hemorrhage finally ensues ; during the first three months, a probable diagnosis only can be made. A positive diagnosis of rupture is made by pain and collapse with profound anæmia. The child is usually not considered in the question of prognosis or operation ; the prognosis for the mother, when the cases proceeded without operative interference, he found to be 68.8 per cent. mortality and 31.2 per cent. recovery. In 585 cases in which operation was performed, 76.6 per cent. recovered and 23 per cent. perished. Martin had enlarged Schauta's table and found that in 265 cases treated expectantly, 36.9 per cent. recovered, and in 515 cases operated upon, 76.7 per cent recovered. His belief is that operation is invariably indicated. The morphine treatment of Winckel is not indorsed, nor the treatment by electricity ; the ovum should be completely removed ; when the fœtal sac cannot be completely extirpated, it may be stitched to the abdominal wall or punctured and drained through the vagina.—*Am. Jour. of Med. Sciences.*

